# Dermoscopy of nasal and auricular gouty tophi^⋆^

**DOI:** 10.1016/j.abd.2022.12.014

**Published:** 2024-04-22

**Authors:** Bruno Simão dos Santos, Maria Augusta Pires Maciel, Neusa Yuriko Sakai Valente

**Affiliations:** Service of Dermatology, Hospital do Servidor Público Estadual, São Paulo, SP, Brazil

Dear Editor,

A 62-year-old male hypertensive patient, a former alcoholic, suffering from gout for approximately 20 years and undergoing irregular treatment with allopurinol and colchicine, presented with a firm and painless nodular lesion on the nasal dorsum for one year, which progressed with ulceration. On dermatological examination, yellowish papules on the ear helices (Fig. 1) and increased volume in the joints of the hands, elbows, knees and feet were also observed. Dermoscopy of the nasal lesion showed a central amorphous white area, and yellowish areas interspersed with shiny white polymorphic structures on the periphery of the lesion, in addition to diffuse erythema and peripheral branched vessels (Fig. 2). Dermoscopic examination of the helix lesions showed, predominantly, aggregated yellowish-white globular structures (Fig. 3), with branched vessels crossing the lesion and on its periphery (Fig. 3A). In other lesions of the right helix, unlike the previous findings, an amorphous yellowish-white area was observed (Fig. 4A) or an amorphous yellowish-white background with blurred branched vessels scattered over the lesion (Fig. 4B-C). Also in the same location, a lesion with an amorphous white area, a yellowish center and peripheral diffuse erythema could be observed, similar to the nasal lesion (Fig. 4D). The laboratory tests showed the patient had anemia and elevated inflammatory markers, reduced renal function and elevated serum uric acid levels (7.8 mg/dL, RV: 3.5‒7.2 mg/dL). However, urinary uric acid was within normal range (378.4 mg/24h ‒ RV: 250 to 750 mg/24h). Histopathological examination of the lesion on the nasal dorsum showed amorphous or crystalloid eosinophilic deposits in the dermis with a needle-like appearance, corresponding to aggregates of monosodium urate crystals, surrounded by a granulomatous inflammatory infiltrate, compatible with the diagnosis of gouty tophus (Fig. 5).

**Figure 1:**
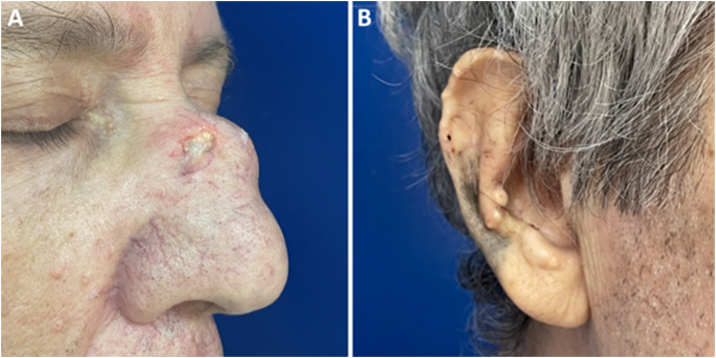
Clinical aspect of gouty tophi. Ulcerated nodular lesion on the nasal dorsum (A) and yellowish papules on the right ear (B).

**Figure 2:**
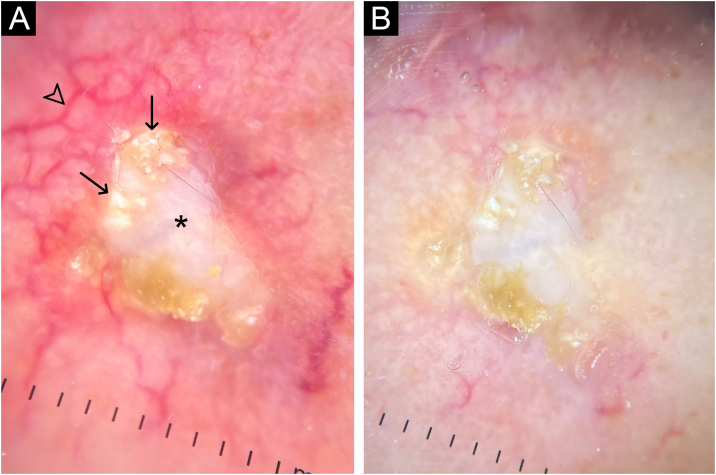
Dermoscopy with polarized light (A) and (B) of the nasal lesion. Central amorphous white area (asterisk), with yellowish areas interspersed with shiny white polymorphic structures (arrows) on the periphery of the lesion, diffuse erythema and blurred branched vessels (arrowhead) best seen in (A) due to the lack of contact between the dermatoscope and the skin. Original magnification, ×10.

**Figure 3:**
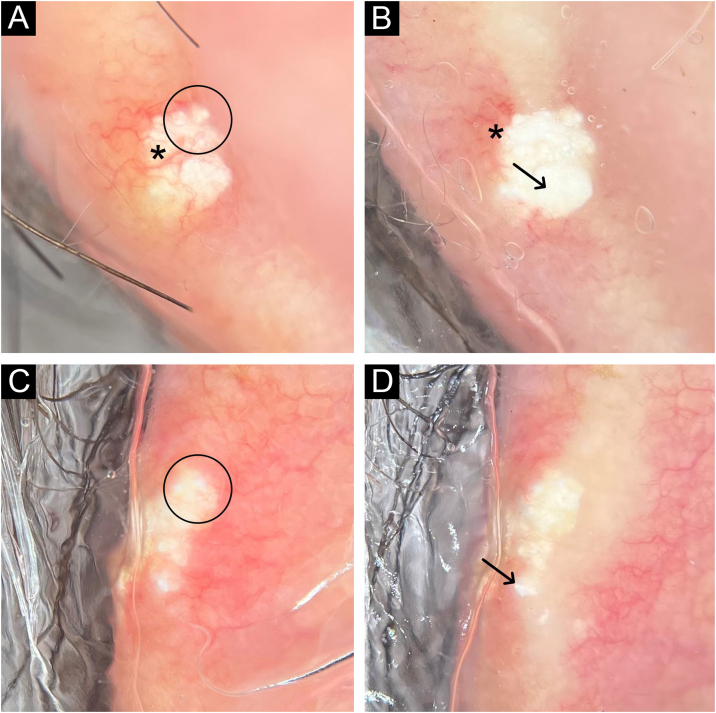
Dermoscopy with polarized light (A to D) of two lesions on the helix. Aggregated yellowish-white globular structures (circles), with branched vessels (asterisks) crossing the lesion (A) and on its periphery (A and B). In the lesion shown in (C) and (D) there are no vessels over the lesion. Shiny white structures can be seen in both lesions (arrows). Original magnification, ×10.

**Figure 4:**
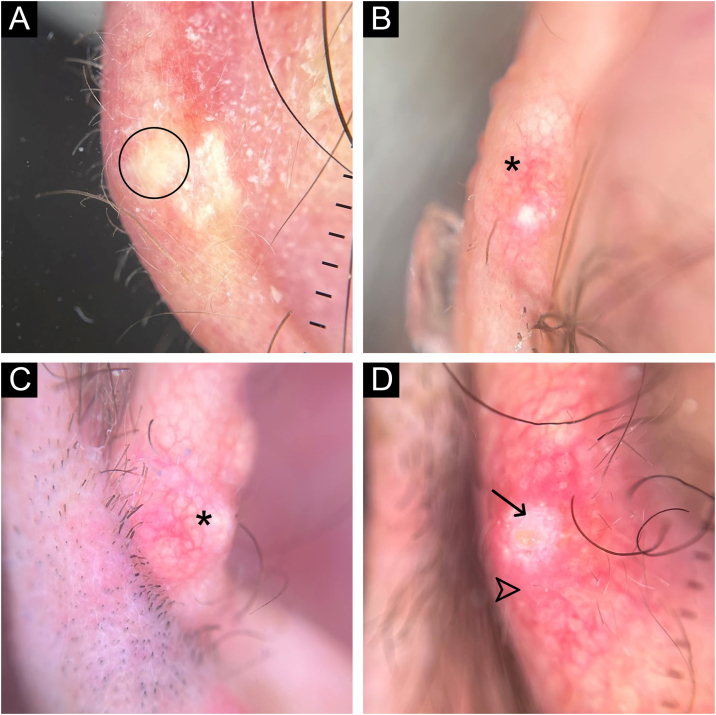
Polarized light dermoscopy without contact with the helix lesions. In (A), amorphous yellowish-white area without vessels (circle); (B) and (C), yellowish-white background with blurred branched vessels over the lesion (asterisks); (D), amorphous white area (arrow) with a yellowish center and peripheral diffuse erythema (arrowhead). Original magnification, ×10.

**Figure 5:**
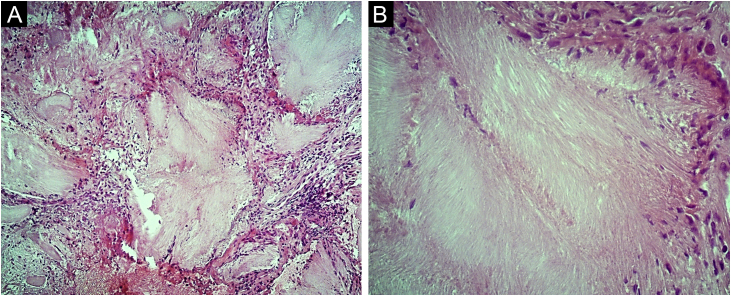
Photomicrographs of the histopathology of the nasal dorsum lesion. Eosinophilic, amorphous or crystalloid deposits in the dermis, surrounded by a granulomatous inflammatory infiltrate. Hematoxylin & eosin, ×100 (A) and ×400 (B).

## Discussion

Gout is the most common inflammatory arthritis and is caused by the deposit of monosodium urate crystals in the joints.^1^ Gouty tophus, the accumulation of these crystals in soft tissues, is the characteristic clinical manifestation of advanced disease but may be the first clinical sign in some cases.^2^ On the skin, it is characterized by firm papules and nodules, with a smooth or multilobulated outline, normochromic, yellowish, or erythematous, which may be ulcerated. The most common locations include the first and fifth metatarsophalangeal joints and the hand and wrist joints. The presentation of gout in the head and neck region is uncommon. The nasal region is usually a rarely affected area.^3^, ^4^, ^5^

Regarding the dermoscopy of gouty tophus, Yoshida et al. reported the dermoscopic findings of an ulcerated gouty tophus on the right toe, describing the presence of whitish structures similar to “horns”, with some shiny dots.^6^ In the present case, grouped amorphous and globular white and yellowish-white areas were observed, associated with several shiny white structures of different shapes, observed both on polarized and non-polarized light. It is possible that such shiny structures correspond to accumulations of monosodium urate crystals located more superficially in the skin. Moreover, the lesions showed different dermoscopic findings compared to those reported in the previous published study.

The diagnosis of gouty tophus, in general, is based on clinicopathological correlation and there are few reports describing the dermoscopic findings of this clinical manifestation. Over the last few years, several studies have shown that dermoscopy can be useful in assisting the non-invasive diagnosis of various inflammatory and infectious diseases.^7^, ^8^, ^9^ Therefore, knowledge of the dermoscopic structures present in gouty tophi becomes relevant, as it can help in the differential diagnosis of dermatoses with a similar clinical picture, such as malignant neoplasms and other metabolic and storage diseases.

## Financial support

None declared.

## Authors’ contributions

Bruno Simão dos Santos: Design and planning of the study; drafting and editing of the manuscript; critical review of intellectual content; effective participation in research orientation; intellectual participation in the propaedeutic and/or therapeutic conduct of the studied case; critical review of the literature; approval of the final version of the manuscript.

Maria Augusta Pires Maciel: Drafting and editing of the manuscript; data collection; intellectual participation in the propaedeutic and/or therapeutic conduct of the studied case; critical review of the literature; approval of the final version of the manuscript.

Neusa Yuriko Sakai Valente: Critical review of intellectual content; effective participation in research orientation; intellectual participation in the propaedeutic and/or therapeutic conduct of the studied case; approval of the final version of the manuscript.

## Conflicts of interest

None declared.
